# Anti-Depressant-Like Effect of Kaempferitrin Isolated from *Justicia spicigera* Schltdl (Acanthaceae) in Two Behavior Models in Mice: Evidence for the Involvement of the Serotonergic System

**DOI:** 10.3390/molecules191221442

**Published:** 2014-12-19

**Authors:** Julia Cassani, Ana María Dorantes-Barrón, Lilian Mayagoitia Novales, Guadalupe Alva Real, Rosa Estrada-Reyes

**Affiliations:** 1 Departamento de Sistemas Biológicos, Universidad Autónoma Metropolitana Unidad Xochimilco, Mexico D.F. 04960, Mexico; E-Mail: casnij@hotmail.com; 2 Laboratorio de Fitofarmacología, Dirección de Investigaciones en Neurociencias, Instituto Nacional de Psiquiatría Ramón de la Fuente Muñiz, Mexico D.F 14370, Mexico; E-Mail: danna196799@hotmail.com; 3 Departamento de Etología, Dirección de Investigaciones en Neurociencias, Instituto Nacional de Psiquiatría Ramón de la Fuente Muñiz, Mexico D.F, 14370, Mexico E-Mail: mayagn@imp.edu.mx; 4 Subdirección de Investigaciones Clínicas del Instituto Nacional de Psiquiatría Ramón de la Fuente Muñiz Instituto Nacional de Psiquiatría Ramón de la Fuente Muñiz, Calzada Mexico-Xochimilco 101, Col. San Lorenzo Huipulco, Delegación Tlalpan, Mexico D.F 14370, Mexico; E-Mail: real474@imp.edu.mx

**Keywords:** antidepressant, muitle, *Justicia spicigera*, serotonergic actions, kaempferitrin

## Abstract

We evaluated the antidepressant-like effect of kaempferitrin (Km) isolated from the plant *Justicia spicigera* (Asteraceae), which is used in traditional medicine for relieving emotional disorders, such as “la tristeza” (sadness or dysthymia) and “el humor” (mood changes). The actions of Km were evaluated in a forced swimming test (FST) and a suspension tail test (TST) in mice. We explored the involvement of the serotonergic system and the hypothalamic-hypophysis-adrenal axis (HPA) in the antidepressant-like effect of Km. To evaluate nonspecific effects of Km on general activity, the open field test (OFT) was performed. Km at 5, 10, and 20 mg/kg induced an antidepressant-like effect. Sub-effective dose of Km (1 mg/kg) produced a synergistic effect with imipramine (6.25 mg/kg) and fluoxetine (10 mg/kg) but not with desipramine (3.12 mg/kg). Pretreatment with *p*-chlorophenylalanine methyl ester (PCPA), a serotonin synthesis inhibitor, *N*-{2-(4-(2-methoxyphenyl)-1-piperazinyl}-*N*-(2-pyridinyl)cyclohexecarboxamide (WAY-100635), a selective 5-HT_1A_ receptor antagonist, and 8OH-DPAT, a selective 5-HT_1A_ agonist, but not pindolol (10 mg/kg) blocked the anti- immobility effect induced by Km. Taken together, these results indicate that the antidepressant-like effect of Km is related to the serotonergic system, principally 5-HT_1A_. This effect was not related to changes in locomotor activity.

## 1. Introduction

Depression and anxiety disorders represent the second most common health problems worldwide [1,2,3,4]. Published data suggest that the current lifetime prevalence for depression is as high as 21% in the world population [5]. Comorbid depression and anxiety are highly prevalent conditions. Patients with panic disorder, generalized anxiety disorder, social phobia and other anxiety disorders frequently also present with depressive disorder. Approximately 85% of patients with depression also experience significant symptoms of anxiety. Similarly, comorbid depression occurs in up to 90% of patients with anxiety disorders [6].

The relationship between depression and anxiety disorders has been a matter of controversy for long time. Recent evidence suggests genetic and neurobiological similarities between depressive and anxiety disorders. Early recognition of comorbid conditions is key to the successful treatment of patients with mixed depressive and anxiety disorders. Antidepressant medications, including selective serotonin reuptake inhibitors, tricyclic antidepressants and monoamine oxidase inhibitors, are highly effective in the management of comorbid depression and anxiety [7]. However, the search for better anxiolytic and anti-depressive drugs is ongoing, and natural compounds have historically been an important source of several clinically useful anti-depressive agents [8]. 

Medicinal therapies with plants may be an effective alternative for the treatment of anxiety and depressive disorders. These natural resources have also proven to be an inexhaustible source of bioactive molecules [9,10,11].

*Justicia spicigera* Schltdl belongs to the Acanthaceae family and has several common names. It is variously known in Mexico as “muicle, hierba de limalin (limali´s grass), muhuite, expaxihuitl, moyotle, moyotli, trompetilla (trumpet), and huitzixochitl. Among the Mayan ethnic group it is known as “*ych-kaan*”, but the most common names are “hierba azul and hierba tinta” (ink grass or blue grass) due to the dark color of its aqueous preparations, ranging from red to dark blue. Among the Totonac community of Tuzamapan de Galeana in the Puebla state of Mexico, *J. spicigera* is known as “moitle” or “muicle” and “limanin” and it is employed as emmenagogue and as a protection against an illness known as “alferecia” (sobbing spasms), and against “limpias” or “levantada de sombra” (shadow lifting) to ward off the sadness of the loss souls [12,13]. This plant is also used for its antialgesic, antidiabetic, anti-inflammatory, and anti-seizure properties, as well as a “nerve tonic” [12,14]. Decoctions of *J. spicigera* are used for the treatment of emotional symptoms associated with the menopause, such as moodiness, sadness, and irritability. *J. spicigera* also relieves peri-menopausal phase symptoms [15]. Additionally, other species of *Justicia* such as, *J. pectoralis* and *J. procumbens* are also used for their central nervous system (CNS) effects as sedative agents and as treatments for epilepsy and other mental disorders and headaches [16,17].

The presence of free flavonoids and polar flavonoid glycosides in several species of the *Justicia* genus, including *J. spicigera* [18,19] has been described. Flavonoid family compounds have diverse CNS effects; for example, 3-*β*-O-flavanol rutinosides are anxiolytic and sedative: quercetin-3-*β*-O- rutinoside, rutin, or derivates of 7-*β-O*- rutinoside, such as linarin [20,21] and flavanone 7-*β-O*- rutinoside such as neoponcirin and hesperidin [22,23]. However, the antidepressant effects of flavonoids have not been widely explored.

Numerous studies have found that depression is related to alterations in the central serotonergic system and to altered activity of the hypothalamic-pituitary-adrenal axis (HPA). It is assumed that serotonin stimulates HPA axis activity, and drugs that enhance brain serotonergic function increase corticotrophin-releasing, adrenocorticotropic hormone, and cortisol secretion in controls and depressed subjects, it has been that some antidepressant drugs, are able suppresses cortisol secretion, contributing to regulation of HPA axis activity [24,25,26,27,28,29].

The aim of the current study was to isolate kaempferitrin (Km), the main secondary metabolite of a hydro-alcoholic extract from the *Justicia spicigera* (Asteraceae) plant, and evaluate its antidepressant-like effects in two predictive paradigms of depression in mice, the tail suspension (TST) and forced swimming tests (FST). We also explored the possible neurotransmission pathways that underlie the antidepressant-like actions of Km using synergism with sub effective doses of antidepressant drugs, such as imipramine (IMI), fluoxetine (FLX), and desipramine (DMI) in combination with non-effective doses of Km. In independent experiments, Km was co-administered with drugs that affect 5-HT neurotransmission, such as WAY-100635, PCPA and pindolol. Finally, is well known that there is a dysregulation of the hypothalamic-hypophysis-adrenal axis (HPA) activity in patients with depressive disorders. On these bases, we hypothesize that, if serotonergic system is involvement in the Km action, then Km could diminish the corticosterone high levels induced by forced swimming, for archive that we measured serum corticosterone levels of mice treated with those doses of Km were active in the FST.

## 2. Results and Discussion

**Figure 1:**
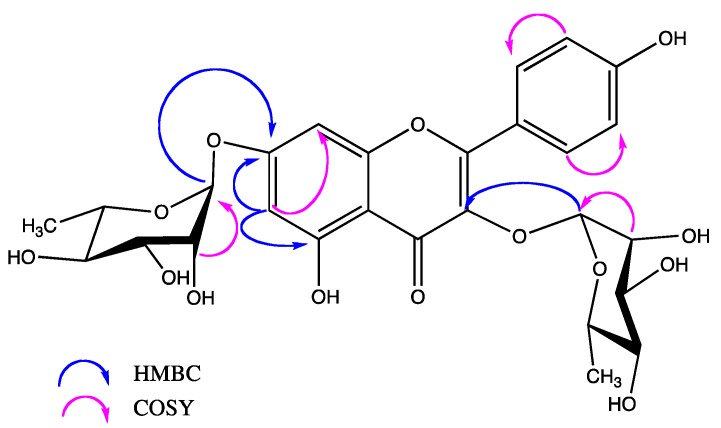
Structure and key COSY and HMBC correlations observed for kaempferitrin.

The aerial part of *Justicia spicigera* was dried, ground and extracted with 70% ethanol at room temperature in a closed container for 72 h. After extensive column chromatography, Kaempferitrin (Figure 1) was isolated as a yellow crystalline solid with a melting point = 198–200 °C, (EtOAc-MeOH recrystallization system, until a constant melting point) and identified by analysis of its NMR and MS spectral characteristics and by comparison with those described in the literature [30,31].

Medicinal therapies using plants may be an effective alternative treatment for anxiety and depressive comorbid disorders. Medicinal plants of the *Justicia* genus, such as *Justicia pectoralis*, are used to treat depression and anxiety disorders [32]. Moreover, *Justicia spicigera* is rich in flavonoid compounds, whose anxiolytic effects have also been described [33,34]. However, the antidepressant potential of these compounds has not been explored.

We decided to evaluate the antidepressant-like effects of kaempferitrin (Km), one of the main constituents of the hydro alcoholic extract of *J. spicigera*, a plant traditionally used for mood and anxiety disorders, in two behavioral models of depression, TST and FST.

TST and FST represent behavior despair models and are widely used for evaluating antidepressant-like activity [35,36,37]. Immobility in the two tests reflects behavioral despair similar to human depression, but each test contributes different information because of the variability in response to certain antidepressants, indicating potentially different substrates and neurochemical pathways mediating performance in the two tests. The TST shows a different spectrum of pharmacological sensitivity from FST [38,39].

**Figure 2:**
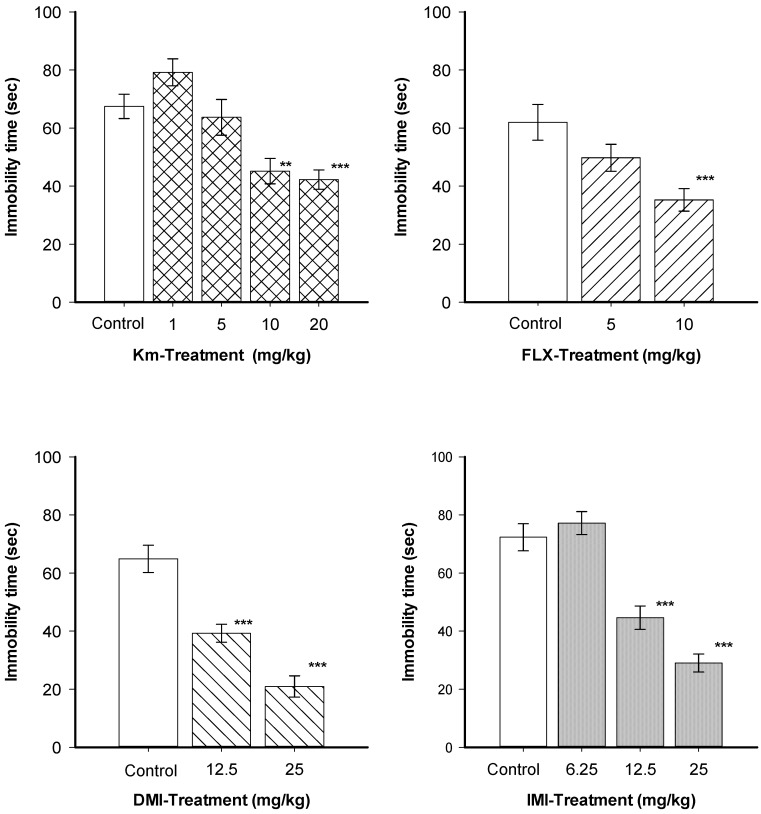
Effects kaempferitrin (Km), fluoxetine (FLX) desipramine (DMI), and imipramine (IMI) on the tail suspension test.

As shown in Figure 2, a single oral administration of Km at 1 and 5 mg/kg did not produce a significant change in the immobility time in the TST, while 10 and 20 mg/kg reduced the immobility time significantly (H = 26.05, df = 4, *p* ≤ 0.001). Statistical analysis revealed that all reference treatments; FLX (H = H = 35.12, df = 2, *p* ≤ 0.001), DMI (H = 19.91, df = 2, *p* ≤ 0.001) and IMI (H = 28.39, df = 3, *p* ≤ 0.001) produced a significant reduction of immobility time in a dose-dependent manner. The TST produces moderate stress in mice, which is reduced by tricyclic and serotonergic antidepressants. Km induced a clear antidepressant-like effect in this model. Moreover, the antidepressant-like effect displayed by Km (10 and 20 mg/kg) was similar to that observed for the positive control drugs IMI, FLX, and DMI.

**Figure 3:**
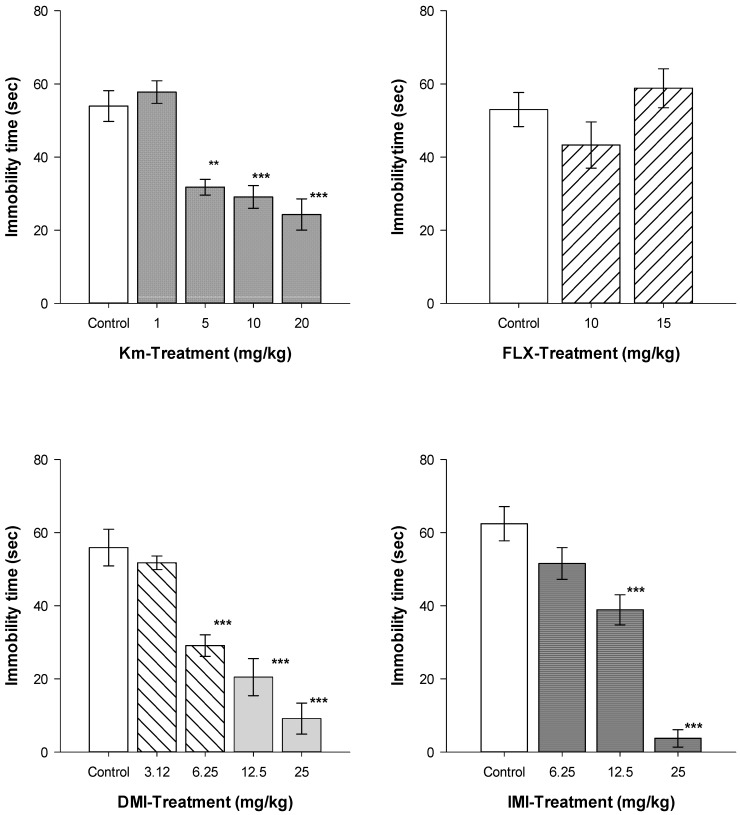
Antidepressant-like effects Km, FLX, DMI, and IMI on the Forced Swimming Test.

As shown in Figure 3, oral treatment with a single dose of Km at 1 mg/kg did not reduce immobility in the FST, while at 5, 10, and 20 mg/kg, a significant reduction in the immobility time was observed. IMI or DMI also produced a significant decrease in immobility time on the FST. Dose-response curves were obtained with IMI (H = 56.13, df = 3, *p* ≤ 0.001), DMI (H = 29.11, df = 4, *p* ≤ 0.001) and Km (H = 20.75, df = 3, *p* ≤ 0.001). In contrast, FLX treatment did not produce significant differences in immobility time (H = 0.923, df = 2, *p* = 0.630) in comparison to the control group. The stress induced by the FST is stronger than that produced by the TST, and a single dose of SSRIs such as fluoxetine is not able to reduce the immobility behavior produced during the FST [40,41].

Effects of treatments with single doses oral route of kaempferitrin (Km) at a 1, 5, 10 and 20 mg/kg and single treatment with fluoxetine (FLX; 5 and 10 mg/kg), desimipramine (DMI; 12.5 and 25 mg/kg), and imipramine (IMI 6.25, 12.5, and 25 mg/kg) by intraperitoneally route in the Tail Suspension Test (TST) in mice. All results are expressed as the average ± SEM of 8 to 12 animals. Comparisons were made using the Kruskal-Wallis analysis of variance based on rank, followed by the Mann-Whitney-U-test: ** *p* < 0.01, *** *p* < 0.001.

Effects of treatment with single doses oral route of Kaempferitrin (Km) at a 1, 5, 10 and 20 mg/kg and single treatment with fluoxetine (FLX; 10 and 15 mg/kg), desimipramine (DMI; 3.12, 6.25, 12.5 and 25 mg/kg), and imipramine (IMI 6.25, 12.5, and 25 mg/kg) by intraperitoneally route in the Forced Swimming Test (FST) in mice. All results are expressed as the average ± SEM of 8 to 12 animals. Comparisons were made using the Kruskal-Wallis analysis of variance based on rank, followed by the Mann-Whitney-U-test: ** *p* < 0.01, *** *p* < 0.001.

Based on these results, the doses 1 and 10 mg/kg were selected as the sub-effective and active dose of Km, respectively, for the subsequent experiments. To ensure that nonspecific effects, such as hypo or hyper ambulatory activity, were not affecting our results, all treatments evaluated in the TST and FST were also analyzed in the open field test (OFT). As shown in Table 1, the tested doses of antidepressants or Km did not affect general locomotor activity (count numbers: H = 10.72, df = 3, *p* = 0.030 and rearing: H = 3.18, df = 4, *p* = 0.52). Even though, a 20 mg/kg, Km produced a slight increase in the count number compared to the control group, with a significance of T = 76.5, *n* = 8, and *p* = 0.014 in the Mann-Whitney U test, it did not affect the exploratory behavior (rearing number). These results indicate that the antidepressant-like actions of Km are not caused by elevated activity rates in experimental animals, in particular at lower doses. These data indicate that the antidepressant-like effects of Km are specific. Additionally, as a first approximation, these results allow us suggest that the antidepressant-like effect of Km could be more effective than fluoxetine, a SSRIs drug, in the FST.

**Table 1:** Effects of Km, FLX, DMI, and IMI on open field test activity.

Treatment (mg/kg)	Ambulatory Activity (Count Number/5 min)	Rearing Number/5 min
Control	31.7 ± 3.24	17.6 ± 1.46
Km1	35.0 ± 3.31	13.0 ± 3.40
Km5	29.0 ± 3.48	20.5 ± 3.90
Km10	32.1 ± 3.64	13.8 ±3.23
Km20	44.7 ± 0.2.29 *	17.7 ± 2.80
	H = 10.72, df = 4, *p* = 0.030	H = 3.18, df = 4, *p* = 0.527
		
Control	41. 12 ±1.32	25.25 ± 3.63
FLX10	37.00 ± 2.17	21.75 ± 3.37
FLX 15	45.37 ± 3.13	28.12 ± 0.81
	H = 5.39, df = 2, *p* = 0.067	H = 3.56, df = 2, *p* = 0.168
Control DMI 3.12 DMI 6.25	37.7 ± 3.24 35.50 ± 5.58 40.5 ± 6.33	29.3 ± 5.73 26.53 ± 3.82 26.8 ± 4.20
DMI 12.5	47.8 ± 3.92	28.0 ± 3.53
DMI25	40.1 ± 4.76	18.7 ± 5.26
	H = 6.08, df = 4, *p* = 0.193	H = 4.97, df = 4, *p* = 0.06
Control	38.8 ± 1.29	25.2 ± 3.63
IMI 6.25	44.5 ± 6.04	36.5 ± 4.67
12.5	30.2 ± 4.43	27.5 ± 2.77
25	41.12 ± 6.18	34.5 ±7.72
	H = 3.508, df = 3, *p* = 0.320	H = 7.38, df = 3, *p* = 0.061

Effects of treatment with single doses oral route of kaempferitrin (Km) at a 1, 5, 10 and 20 mg/kg and fluoxetine (FLX; 10 and 15 mg/kg; i.p.), desimipramine (DMI; 3.12, 6.25, 12.5 and 25 mg/kg, i.p.), and imipramine (IMI 6.25, 12.5, and 25 mg/kg) in the Forced Swimming Test (FST) in mice. All results are expressed as the average ± SEM of 8 to 12 animals. Comparisons were made using the Kruskal-Wallis analysis of variance based on rank, followed by the Mann-Whitney-U-test: * *p* < 0.05.

**Figure 4:**
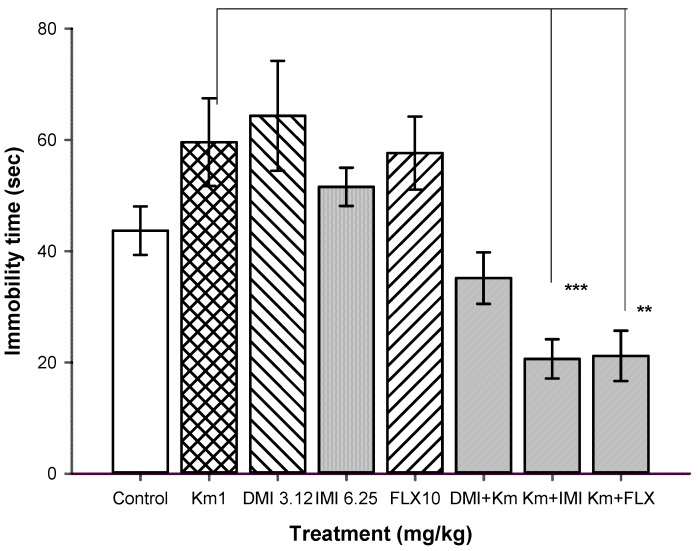
Effect of the combination of Km and IMI, DMI, and FLX on the FST.

To trace the mechanism of the antidepressant-like effect of Km, we conducted synergic experiments, in which Km and one the antidepressant drugs imipramine (IMI), desipramine (DMI) and fluoxetine (FLX) were administered. We evaluated immobility behavior in the FST. Figure 4 shows the results of the combined administration of suboptimal doses of Km (1.0 mg/kg) with sub-threshold doses of IMI (6.25 mg/kg), DMI (3.12 mg/kg), or FLX (10 mg/kg). The combination of Km at 1 mg/kg facilitated the antidepressant effect of a suboptimal dose of IMI, an antidepressant drug that inhibits the reuptake of serotonin [40,41]. Km + IMI produced a significant reduction in the immobility time with respect to the control group (T = 100, *n* = 8, *p* ≤ 0.001). In contrast, the combination of threshold doses of Km and DMI, a selective norepinephrine reuptake inhibitors (SNRIs) and to a minor extent of serotonin [42,43], did not produce significant changes in immobility behavior compared to the control group (T = 81, *n* = 8, *p* = 0.19). Acute doses of SSRIs such as fluoxetine do not have antidepressant-like effects on the FST [40,41,42]. As mentioned above, FLX was not able to reduce immobility time on the FST, which agrees with our results [40,44]. Interestingly, the combination of FLX at 10 mg/kg and a non-effective dose of Km (1 mg/kg) showed evident antidepressant activity (T = 93, *n* = 8, *p* ≤ 0.001) on the FST. This antidepressant-like effect was similar to that produced by FLX in the suspension tail test. The synergistic antidepressant-like effect produced by a sub-effective dose Km and FLX was an interesting result that may derive from the effect of Km in the FST and an interaction with a 5-HT receptor subtype. Locomotor activity tests were performed to eliminate the possibility of false positive results. None of the combinations tested in the FST produced significant changes in locomotor activity (data not shown).

Effect of a non-effective dose of Km (1 mg/kg), DMI (3.25 mg/kg), IMI (6.25 mg/kg) and FLX (10 mg/kg) given alone and Km (1 mg/kg) given in combination with DMI, IMI or FLX on immobility time in the FST. All results are expressed as the average ± SEM of 8 to 12 animals. Comparisons were made using the Kruskal-Wallis analysis of variance based on rank, followed by the Mann-Whitney-U-test: ** *p* < 0.01, *** *p* < 0.001.

A large number of experimental and clinical studies indicate that the serotonin (5-HT) system is strongly implicated in the regulation of mood and anxiety. Several pieces of evidence have implicated abnormalities in serotonergic neurotransmission in the pathophysiology of depressive disorders. The enhancement of 5-HT neurotransmission underlies the therapeutic response of various types of antidepressant treatments [41,45,46].

In accordance with our findings earlier, we explored the involvement of the 5-HT system in the antidepressant-like effect of Km. We depleted 5-HT by the tryptophan hydroxylase inhibitor *p*-chlorophenylalanine (PCPA, 100 mg/kg, i.p., four days) before treatment with Km. PCPA blocked the anti-immobility effect of Km on the FST. As shown in Table 2, Km treatment at 10 mg/kg produced a significant reduction on immobility time (T = 96, *n* = 8, *p* = 0.002), while pretreatment with PCPA prevented the anti-immobility effect of Km. The group treated with Km *plus* PCPA did not show a significant difference with respect to the control group. (T = 61.50, *n* = 8, *p* = 0.52), while, when compared with PCPA alone was not difference (T = 80.0, *n* = 8–11, *p* = 0.967).

PCPA is able to deplete almost 80% of the endogenous serotonin in the rodent brain without affecting NA or DA levels [46,47,48]. Our results suggest the involvement of the serotonergic system in the antidepressant-like actions of Km, and show that endogenous serotonin in the synaptic cleft plays an important role in the antidepressant-like action of Km. These findings further indicate the participation of presynaptic 5-HT_1A_ receptors. However, we cannot rule out the involvement of postsynaptic 5-HT_1A_ receptors in its effect. 

The participation of 5-HT receptor subtypes in the antidepressant-like effect of Km was also analyzed. We attempted to block the effect of Km on the FST with the antagonists pindolol and WAY100635 and to increase the effect of Km with the 5-HT_1A_ agonist, *8OH*-DPAT [48,49]. 

Mice were treated with Km at 10 mg/kg in combination with pindolol (10 mg/kg), a 5-HT_1A/1B_ receptor/*β*-adrenoreceptor antagonist. As shown in Table 2, rather than block the anti-immobility behavior induced by Km, pindolol produced a facilitation of Km, producing a significant reduction in the immobility time compared to Km alone (T = 69, df = 8, *p* ≤ 0.001). Several studies have shown that the co-administration of pindolol with SSRIs such as FLX reduces the time to onset of the clinical effects of SSRIs [49,50,51]. The SSRIs increases the concentration of 5-HT in the synaptic cleft by preventing its reuptake, which leads to the activation of 5-HT_1A_ receptors [49,50,51].

Pindolol could act by preventing the activation of 5-HT_1A_ elicited by SSRIs and other 5-HT drugs, hence reducing negative feedback and resulting in increased 5-HT neural cell firing, as well as increasing 5-HT content. Pindolol is also a β-adrenergic inhibitor, and it does not modify the actions of Km [50,51,52,53]. These facts support the idea of a predominant involvement of the serotonin system in the antidepressant-like effects of Km, and suggest that the noradrenergic system plays a secondary role. However, specific experiments would be necessary to determine the possible involvement of the noradrenergic system in the actions of Km. 

Stronger evidence for the involvement of 5-HT_1A_ receptors in the antidepressant-like effect of Km came from the finding that the selective 5-HT_1A_ receptor antagonist WAY 100635 was able to completely block the anti-immobility effect of Km in the FST. As shown in Table 2, the selective 5-HT_1A_ receptor antagonist WAY100635 (0.03 mg/kg, s.c.), per se has no effect while, pretreatment of mice with WAY100635 (0.03 mg/kg, s.c.), blocked the anti-immobility behavior elicited by Km (10 mg/kg) in the FST, (T = 69.0, *n* = 8, *p* = 0.958) suggesting that the antidepressant-like effect of Km is mediated through this receptor subtype. It’s important to point that none of the drugs combinations modified the locomotor activity of test animals (data no showed) when were tested in the OFT.

Moreover, sub-effective doses of Km in combination with non-effective *8OH*-DPAT (0.05 mg/kg) treatment produced a synergistic antidepressant-like effect in the FST. *8OH*-DPAT has been reported to reduce immobility time in the FST in mice. However, data from the literature are rather conflicting regarding the site and mechanism of action of *8OH*-DPAT. Activation of presynaptic 5-HT_1A_ receptors in the dorsal raphe by *8OH*-DPAT was initially proposed to explain its effect in the FST. Together, our results regarding the involvement of 5-HT_1A_ receptors in the antidepressant-like effect of Km in the FST strongly indicate that it somehow interacts with these receptors, possibly as a partial 5-HT_1A_ receptor agonist [49,50,51,52,53,54].

**Table 2:** Effect of PCPA, pindolol, WAY100635 and 8OH-DPAT on the immobility time of Km in FST of mice.

Treatment 1 (mg/kg)	*plus*	Treatment 2 (mg/kg)	Immobility Time (sec)
**Experiment I**			
Vehicle		vehicle	57.93 ± 2.02
**Vehicle**		**Km (10)**	27.73 ± 3.62 **
Pcpa (100)		*vehicle*	50.36 ± 4.52
Pcpa (100)		Km(10)	51.26 ± 6.24
			**H = 10.246, df = 3, *p* = 0.006**
**Experiment II**			
Vehicle		vehicle	57.93 ± 2.02
Pindolol (10)		Vehicle	59.86 ± 7.83 ^###^
Vehicle		Km (10)	29.11 ± 3.08 ***
Km (10)		Pindolol (10)	13.10 ± 2.42 ***^, ###^
			**H = 25.77, df = 3, *p* ≤ 0.001**
**Experiment III**			
Vehicle		vehicle	52.93 ± 2.02
Way100635 (0.03)		vehicle	58.03 ± 5.49
Vehicle		Km (10)	29.11 ± 3.08 ***
Way(0.03)		Km(10)	54.24 ± 6.85
			**H = 14.02, df = 3, *p* = 0.003**
**Experiment IV**			
Vehicle		vehicle	58.13 ± 2.31
Vehicle		Km (1)	60.07 ± 3.82
*8OH*-DPAT (0.05)		vehicle	60.85 ± 3.85 ^###^
*8OH*-DPAT (0.5)		vehicle	30.78 ± 3.48 ***
*8OH*-DPAT (0.05)		Km (1)	34.91 ± 3.48 ***^, ###^
			**H = 30.59, df = 5, *p* = 0.001**

Effects of joint administration of Km and PCPA (100 mg/kg daily, during the 4 days previous to the test; i.p. route), Km (oral route) *plus* pindolol (i.p.; 10 mg/kg) 30 min before the FST, WAY 100635 at 0.03 mg/kg (s.c.via), 30 min before Km (10 mg/kg) administration and 60 min before the FST. of Km and *8OH*-DPAT, were joint administered 30 min before the start of the FST; All results are expressed as the means ± standard error (*n* = 8–14). Comparisons were made using a Kruskal-Wallis analysis of variance based on rank, followed by the Mann-Whitney-U-test: ** *p* < 0.01, *** *p* < 0.001 when compared with control group and ^###^ *p* < 0.001 when compared with drug alone: PCPA (100) *vs.* [PCPA (100) *plus* Km (10)]: T = 80.0, *n* = 8–11, *p* = 0.967; Pindolol (10) *vs.* [Pindolol (10) *plus* Km (10)]: T = 124.0, *n* = 8, and *n* = 11, *p* ≤ 0.001; WAY100635 *vs.* [WAY100635 *plus* Km (10)]: T = 69.0, *n* = 8, *p* = 0.958; *8OH*-DPAT (0.05) *vs.* [8OH-DPAT (0.05) plus Km (1)]: T = 98.0, *n* = 8–12, *p* ≤ 0.001.

Finally, several studies have found that mood disorders such as depression are related to altered activity of the hypothalamus-pituitary-adrenal axis (HPA), producing abnormally increased levels of cortisol [52,54,55]. Moreover, it is known that major depression is frequently associated with HPA hyperactivity. Clinical studies have shown that depressed patients have an increased cortisol concentration in the plasma, as reflected by an abnormal 24-h pattern of cortisol secretion [52]. Normalization of the HPA axis was hypothesized to play an important role in mediating antidepressant activity [25,26,27]. We explored the possible that Km may influence HPA activity. The training section (pre-test) produced a significant increase in serum corticosterone levels compared with the mice not exposed to the FST (naïve *vs.* vehicle-treated group: *t* = 2.13, *p* < 0.04). This is consistent with previous studies that have been demonstrated that swim stress is a potent activator of the HPA axis, causing corticosterone release into the blood [28,29,55,56].

As shown in Table 3, Km at 10 and 20 mg/kg or IMI at 25 mg/kg were not able to prevent the elevation of corticosterone generated during the pre-test session of the FST (F_(4,24)_ = 4.96, *p* > 0.05).

**Table 3:** Corticosterone serum levels of mice treated with vehicle, Km (10 and 20 mg/kg), and IMI.

Treatment (mg/kg)	Corticosterone Levels (nmol/L)
Naïve	495.42 ± 58.98
Saline solution	891.42 ± 141.13 ***
Km 10	1000.52 ± 38.42 ***
Km 20	1271.51 ± 200.91 ***
IMI 25	925.25 ± 128.59***
	F_(4,24)_ = 4.96, *p* = 0.05

Effect of the exposure to the pre-test session of the FST in naive (no treatment), vehicle-treated mice, Km (10 and 20 mg/kg; oral route) and IMI (25 mg/kg; i.p. via) treated mice on serum corticosterone levels 24 h after the pre-test. All results are expressed as the average ± S.E.M., of independent groups of eight animals Treatments *vs.* naive group: *** *p* ≤ 0.05.

The HPA axis in mice exposed to the FST was not sensitive to Km acute treatment. A possible explanation could be that to produces a decrease in corticosterone levels, sub-acute or chronic treatment of Km may be necessary.

Another possibility is that antidepressant actions could reduce corticosterone levels below the optimum levels, and thus the HPA axis is activated to produce the corticosterone necessary to maintain the homeostasis of system. If this were the case, what we measured is the increase in the production of corticosterone. However, more experiments are needed to verify these possibilities. The evaluation of the chronic effects of Km on HPA axis activity is being ongoing in our laboratory.

Together, these results demonstrate that Km induces a clear antidepressant-like effect, without producing disturbances in ambulatory activity of the experimental animals. Additionally, our results at least partly show that the serotonergic neurotransmitter system is involved in the actions of Km. However, is necessary carry out more experiments to determine the specific mechanisms underlying the actions of Km.

## 3. Experimental

### 3.1. General Information

The solvents used in this work were purchased from Aldrich Chemical Co., Inc. (Milwaukee, WI, USA). Flash column chromatography (FCC) was performed using flash silica gel (32–63 μm), and a solvent polarity correlated with TLC mobility was employed. The chromatographic columns were monitored by TLC carried out on 0.25 mm Merck silica gel plates. Developed TLC plates were visualized under a short-wave UV lamp and by heating plates that were dipped in ethanol/H_2_SO_4_ (15:1). Melting points, determined with a Fisher apparatus, are uncorrected. NMR experiments were conducted on an Agilent 600 MHz machine with One NMR Probe (Santa Clara, CA, USA). Instrument chemical shifts are reported in ppm with respect to TMS (tetramethylsilane) and the coupling constant (*J*) is reported in Hz.

### 3.2. Plant Material

The aerial parts of *Justicia spicigera* (Acanthaceae) were collected by Biologist Guadalupe Alva Real in the “Maguey blanco” community (Latitude: +20°25′13.36″ and longitude −99°10′1.84″) in the municipality of Ixmiquilpan, Hidalgo State, México on 5 May 2008. The botanical and taxonomic identification was carried out by Francisco Marchena. A sample of this species was deposited at the Herbarium of the Escuela Nacional de Antropología e Historia (ENAH) calle Zapote s/num y Periférico Sur, Col Isidro Fabela, Del Tlalpan, Mexico, DF, Mexico. Voucher number 3581.

### 3.3. Isolation, Purification and Chemical Structure Determination of Kaempferitrin

Dried ground aerial parts of *J. spicigera* (5 kg) were macerated with 70% ethanol for approximately 72 h. The extract was obtained and the solvent was evaporated to dryness under vacuum. The ethanol extract was subjected to separation and isolation procedures. An aliquot of the ethanol extract was subjected to phytochemical analysis screening using standard procedures. The TLC profile showed that the ethanol extract is a complex mixture of compounds. A phytochemical analysis by the color test and thin layer chromatography (TLC) showed the presence of flavonoids, polyphenolic compounds detected by the Folin-Ciocalteu’s reagent, terpenoids, saponins, and tannins. The extract was negative for anthraquinones and alkaloids. The anthocyanin test was positive. The ethanol extract was susceptible to pH changes, showed a dark blue color in acid solution and an intense red color in alkaline solution, confirming the presence of anthocyanin type compounds.

The ethanol extract (800.5 g) was washed with hexane and filtered, producing a dark brown viscous mass that was separated by CC. The chromatography process was performed using an open column packed with 60 G F_254_ Merck silica gel at a proportion of 1:15 with respect to the dry extract. Elution began with hexane followed by mixtures of hexane and EtOAc, EtOAc, mixtures of EtOAc and MeOH with increasing polarity, and MeOH. Fractions of 100 mL were collected and those with similar TLC profiles were combined. A pool from the fractions eluted with EtOAc-MeOH (9:1) was separated by flash chromatography, yielding kaempferitrin (kaempferol-3, 7-*O*-(*R*)-dirhamnoside) (1025 mg), which was purified by successive recrystallizations until the compound produced a constant melting point. The identity of Km was determined by NMR and MS data.

### 3.4. Animals

Adult male Swiss Webster mice were obtained from the vivarium at the National Institute of Psychiatry, Ramón de la Fuente Muñiz and housed eight mice per cage in a room with an inverted light:dark cycle (12:12 h, lights on at 22:00 h) and with free access to water and commercial chow (Purina rodent laboratory mouse chow 5001). All behavioral evaluations were performed between 10:00 and 14:00 h. Animal care was performed in accordance with the general principles of laboratory animal care (Guide for the Care and Use of Laboratory Animals, NIH publication No. 85-23, revised in 1985) [57] and to the “Norma Oficial Mexicana’ (Norma Oficial Mexicana” (NOM-062-ZOO-1999). The experimental protocol was approved by the local ethical committee (NC093620.0).

### 3.5. Drugs

All drugs in this study were administered in a total volume of 10.0 mL/kg body weight. Km was dissolved in 5% *β*-cyclodextrin hydrate in water solution and administered orally. Control animals received the same volume of the vehicle orally; IMI, DMI, FLX; PCPA, pindolol, and *8OH*-DPAT were dissolved in saline solution and administered by intraperitoneally via, and WAY100635 was dissolved in saline and administered by subcutaneous (s.c.) via. 

### 3.6. Tail Suspension Test (TST)

Acoustically and visually isolated mice were suspended 50 cm above the surface of a wooden box by adhesive tape placed approximately 1 cm from the tip of the tail. Each mouse was suspended for a total of 6 min, and long-lasting immobility was recorded during the final 4 min of the test. Immobility behavior was scored only when the mouse remained passively hung and completely motionless.

In independent experiments, we investigated whether a single administration of Km could produce antidepressant-like effects in the TST. A total of fifteen independent groups of eight to twelve mice per group were distributed as follows: four groups received Km at 1.0, 5.0, 10 and 20 mg/kg doses, 30 min before the test. Seven groups as positive control were treated with FLX at 10 or 15 mg/kg, DMI at 12.5 or 25 mg/kg, and IMI at 6.25, 12.5, and 25 mg/kg administered 30 min before the test and another four vehicle-treated groups were used as control group for each of the different treatments [41,42,43,44].

### 3.7. Forced Swimming Test (FST)

Mice were individually placed into glass cylinders (height: 21 cm, diameter; 14.5 cm) containing 15 cm of water at 23 ± 1 °C. All animals were forced to swim for a 15 min period (pre-test), followed by a 3 min session (test) 24 h later. Drugs and Km were administered 30 min before the beginning of the test. The total immobility time was measured in seconds. Immobility behavior was scored when the mouse remained floating and treading water just enough to keep its nose above water. After the swimming sessions, mice were removed from the cylinder and carefully dried, placed in heated cages for 20 min and then returned to their home cages. All experimental sessions were videotaped and later scored by an observer that was unaware of the pharmacological treatments.

Four independent groups of eight to twelve animals each received 1.0, 5.0, 10, and 20 mg/kg doses of Km 30 min before the test. Nine independent groups treated with FLX (10, and 15 mg/kg), IMI (6.25, 12.5, and 25 mg/kg) and DMI (3.12, 6.25, 12.525 mg/kg dose) served as positive controls. Each treatment had its own control group treated with the corresponding vehicle. Drugs were administered 30 min before the test.

### 3.8. Combination of Km with IMI, DMI, or FLX in the FST

To determinate whether Km is able to enhance the effect of antidepressant drugs in the FST, synergism experiments with sub-thresholds doses were designed. Independent groups of mice (*n* = 8–12 per group) were treated with non-effective doses of Km (1.0 mg/kg) in combination with sub-threshold doses of each antidepressant drug according the following schedule: in independent experiments 1.0 mg/kg of Km was administered 30 min before the start of the test, and FLX, IMI or DMI were intraperitoneally administered of 30 min before the start of the test. 

*Experiment I*: A group was treated with Km at a sub-effective dose (1.0 mg/kg) in combination with a sub threshold dose of fluoxetine (FLX; 10 mg/kg), a serotonin reuptake inhibitor, and 30 min after; the mice were tested in the FST.

*Experiment II*: A group of 10 mice was treatment with at a sub-effective dose of Km (1.0 mg/kg) in combination with a sub-optimal dose of DMI (3.12 mg/kg), a norepinephrine reuptake inhibitor reported effective in the FST, and 30 min after were tested in the FST [41,42,43].

*Experiment III*: A group was treated with the combination of sub-effective doses of Km (1.0 mg/kg) plus IMI (6.25 mg/kg) 30 min before being tested in the FST.

### 3.9. Combination of Km with PCPA, WAY 100635, Pindolol, or 8OH-DPAT in the FST

The possible involvement of the serotonergic system in the antidepressant-like effect of Km was explored in the FST. To determinate whether antidepressant drugs are able to block or enhance the antidepressant-like effects of Km in the forced swimming paradigm, experiments to antagonize and synergize the drug were designed. 

*Experiment I*: Two independent groups of eight animals were pretreated with PCPA (100 mg/kg, an inhibitor of serotonin synthesis) and a third group was given only the vehicle, once a day, for four consecutive days [35,47,58,59]. After the last administration of PCPA, one group was treated with Km (10 mg/kg, p.o.) and the other groups only received vehicle. Then, all mice were tested in the FST 30 min later. Another group was treated with acute Km at 10 mg/kg.

*Experiment II*: In a separate set of experiments, independents groups were treated with vehicle, Km (10 mg/kg) or pindolol (10 mg/kg), a 5-HT_1A/B_ receptor/*β*-adrenoceptors antagonist. Another group was treated jointly with 10 mg/kg of Km and 1.0 mg/kg of pindolol, and 30 min after, tested in the FST [37].

*Experiment III*: A total of four independent groups of mice (*n* = 8–10) were employed to explored the ability to WAY 100635 to inhibit the antidepressant-like effect of Km. Two groups were treated with WAY 100635 (0.03 mg/kg) or vehicle, and 60 min later, the FST was carried out. One group was given Km (10 mg/kg) and submitted to the FST 30 min later. The last group received WAY 100635 at 0.03 mg/kg 30 min before Km (10 mg/kg) administration and 60 min before the FST [33,47,48].

*Experiment IV*: We also tested the ability of *8OH*-DPAT (0.05 and 0.5 mg/kg, a 5-HT_1A_ receptor agonist) to enhance the antidepressant-like effects of a sub-threshold Km dose (1 mg/kg) in the FST by the joint administration of Km and *8OH*-DPAT, 30 min before the start of the FST.

Drugs used in these experiments were selected on the basis of literature data and previous results in our laboratory (data not shown), and did not modify locomotor activity [46,47,48].

### 3.10. Open Field Test (OFT)

To eliminate the confounding influence of Km-treatment on locomotor activity, all treatments studied in the antidepressant-like paradigms were analyzed in the open field test. Motor activity was measured in an apparatus consisting of an opaque plexiglass box (40 × 30 × 20 cm) divided into 12 equal squares (11 × 11 cm). The animal was placed into a corner of the cage, and its behavior was videotaped. An observer, who was blind to the pharmacological treatments, registered the total number of times that the animal crossed into square during 5 min (counts/5 min), as well as the number of times the mouse stood on its hind legs (rearing number/5 min). A count is registered when the animal totally crossed from one square to the next. A decrease in the number of counts is considered as a decrease in locomotor activity. The test box was carefully cleaned after each recording. To prevent behavioral changes of the animals after the first experience, mice were tested only once [32,48].

### 3.11. Corticosterone Levels Determination

Corticosterone levels produced by administration of Km at 10 and 20 mg/kg, IMI at 25 mg/kg, vehicle, or free-treatment were measured in serum obtained 30 min after each treatment. Serum corticosterone was quantified using an enzyme-linked immunosorbent assay (ELISA) kit (Enzo Life Science, Inc., Farmingdale, NY, USA, catalog No. ADI-9000-097). The minimum detectable concentration of corticosterone in this assay was estimated to be 0.1 nmol/L. 

An independent group underwent a pre-test session (15 min) and 24 h after were treated with vehicle, IMI at 25 mg/kg or Km (10 and 20 mg/kg). Another group received no treatment (naive) and served as the control group. After 30 min, all the mice were decapitated and blood samples were collected in tubes without anticoagulant. Once clotted, the samples were centrifuged at 3000 rpm at 4 °C for 30 min for serum preparation. These samples were collected and stored at −4 °C until quantification [40,41]. All samples were pretreated with a steroid displacement reagent (included in the kit) to release steroids from carrier proteins [58,60,61].

## 4. Conclusions

Kaempferitrin isolated from the leaves of *Justicia spicigera*, a plant used traditionally, reduced immobility time in the forced swimming test and tail suspension test, without affecting the locomotor activity of mice. To the best of our knowledge, this is the first report about the antidepressant-like effects of kaempferitrin. Kaempferitrin has a clear antidepressant-like effect in mice, which is mediated through the serotonergic neurotransmitter system, mainly through its interaction with presynaptic 5-HT_1A_ receptors. However, we cannot eliminate the involvement of other neurotransmitter systems in the antidepressant-like effects of kaempferitrin. Furthermore, the potentiation of the antidepressant-like effect of fluoxetine by Km might have potential therapeutic value.
